# Repurposed Systemic Pharmacologic Agents in Chronic Pain: Emerging Mechanistic and Clinical Insights

**DOI:** 10.3390/jcm15041572

**Published:** 2026-02-17

**Authors:** Alyssa McKenzie, Rachel Dombrower, Tiffany G. Bittar, Sophia M. McKenzie, Nitchanan Theeraphapphong, Neil Shukla, Hatim Hussain, Alaa Abd-Elsayed

**Affiliations:** 1 School of Medicine, St. Georges University, West Indies 11739, Grenada; 2 School of Medicine, Uniformed Services University of the Health Sciences, Bethesda, MD 20814, USA; 3 Department of Anesthesiology, University of Wisconsin School of Medicine and Public Health, Madison, WI 53715, USA; abdelsayed@wisc.edu

**Keywords:** chronic pain, glucagon-like peptide-1 receptor, sodium–glucose cotransporter-2 inhibitor, cytokines, oxidative stress, analgesics, pathophysiology, inflammation

## Abstract

Chronic pain is a multisystem disorder involving neuroimmune activation, metabolic dysregulation, mitochondrial dysfunction, and alterations in autonomic and sensory signaling, leading to peripheral and central sensitization, reduced responsiveness to standard analgesics, and persistent symptoms. Growing evidence suggests that several widely used systemic drugs, initially developed for metabolic, cardiovascular, immunological, or neurological conditions, interact with biological mechanisms involved in pain pathophysiology. This narrative review examines the mechanistic and emerging clinical evidence describing how systemically administered pharmacological agents interact with pathways implicated in chronic pain, focusing on glucagon-like peptide-1 receptor agonists, sodium–glucose cotransporter-2 inhibitors, metformin, statins, minocycline, ibudilast, low-dose naltrexone, beta-blockers, and cannabinoids. The mechanisms reviewed include glial activation, cytokine signaling, oxidative stress, mitochondrial dysfunction, ion channel sensitization, and autonomic imbalance. The use of these systemic agents may provide additional treatment options for patients with chronic neuropathic, centralized, or mixed pain states who have limited response to conventional therapies, although current clinical evidence remains preliminary.

## 1. Introduction

Chronic pain affects an estimated 20–30% of adults worldwide and contributes to disability, reduced quality of life, and healthcare utilization [1]. Though there have been recent advances in pain classification and expanded treatment options, commonly used pharmacologic therapies, including nonsteroidal anti-inflammatory drugs, gabapentinoids, serotonin–norepinephrine reuptake inhibitors, tricyclic antidepressants, and opioids, often provide limited relief. Their use is further restricted by adverse effects, tolerability issues, and issues regarding long-term toxicity [1,2].

Recent evidence indicates that chronic pain covers a spectrum beyond altered nociceptive transmission to involve coordinated neuroimmune activation, cytokine signaling, mitochondrial dysfunction, and maladaptive central nervous system plasticity [3,4,5]. In this review, chronic pain is discussed primarily in the context of neuropathic pain, nociplastic pain syndromes (including fibromyalgia and other centralized pain states), and inflammatory pain conditions. Additional consideration is given to pain phenotypes associated with metabolic disease, autonomic dysregulation, and neuroimmune activation, reflecting the mechanistic focus of the agents reviewed.

Within this context, drug repurposing has gained interest as a strategy to expand available treatment options for chronic pain [6]. Several agents originally developed for metabolic, cardiovascular, or immunologic indications, including glucagon-like peptide-1 (GLP-1) receptor agonists, sodium–glucose cotransporter-2 (SGLT2) inhibitors, metformin, statins, minocycline, ibudilast, beta-blockers, and low-dose naltrexone (LDN), have demonstrated interactions with neuroinflammatory pathways implicated in chronic pain [6,7]. These agents influence biological processes, including microglial activation, cytokine release, oxidative injury, ion channel sensitization, mitochondrial dysfunction, and autonomic regulation [8]. Their established safety profiles support consideration for use in the management of chronic pain [6].

**Figure 1:**
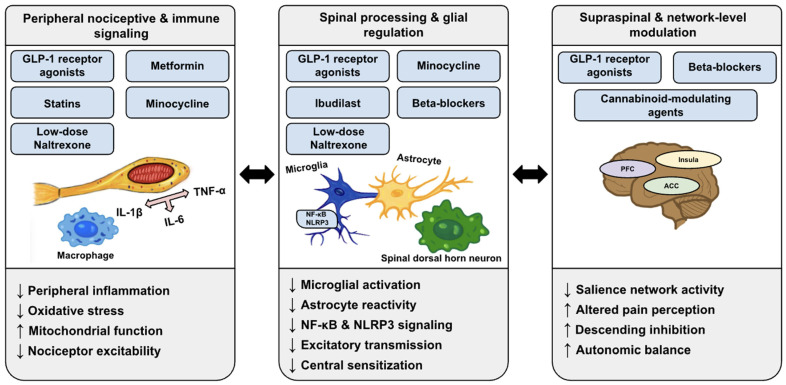
Multisystem model linking chronic pain mechanisms to repurposed systemic pharmacologic agents, in which interacting neuroimmune, metabolic, mitochondrial, autonomic, and sensory processes drive peripheral and central sensitization, and drugs developed for non-analgesic indications converge on shared pathways, including glial activation, cytokine signaling, oxidative stress, mitochondrial dysfunction, ion channel sensitization, and autonomic dysregulation, supporting repurposing in neuropathic, centralized, and mixed pain states.

Although individual agents have been evaluated for their analgesic effects, the current literature does not cover integration of shared mechanisms across these drug classes [8,9]. Improved understanding of how systemically administered repurposed agents modulate neuroimmune, metabolic, and central sensitization pathways is needed to guide translational research and patient selection. Figure 1 provides a conceptual overview of the multisystem mechanisms underlying chronic pain and illustrates how repurposed systemic pharmacologic agents converge on shared neuroimmune, metabolic, mitochondrial, autonomic, and sensory pathways implicated in peripheral and central sensitization.

This narrative review examines mechanistic and clinical evidence related to repurposed systemic pharmacologic therapies for chronic pain from a neuroimmune modulatory perspective. It outlines individual drug biology, summarizes major drug classes with relevant mechanisms, identifies overlapping pathways of action, and considers implications for mechanism-informed analgesic strategies.

To clarify scope and approach, the review focuses on systemically administered pharmacologic agents originally developed for non-analgesic indications that demonstrate mechanistic relevance to chronic pain biology. Emphasis is placed on pathways involving neuroimmune signaling, metabolic and mitochondrial dysfunction, autonomic regulation, and central sensitization, with the aim of linking molecular and cellular effects to reported pain-related outcomes. Evidence discussed includes human studies when available, supplemented by established preclinical models where clinical data are limited.

## 2. Pathophysiologic Rationale: How Neuroimmune and Metabolic Pathways Drive Chronic Pain

Chronic pain involves interactions among neuroimmune, metabolic, mitochondrial, and central nervous system processes, rather than representing a downstream consequence of acute injury [10]. This condition arises from a set of interacting biological mechanisms that affect excitability, immunity, metabolism, and the activity of central networks [3]. These mechanisms alter neuronal excitability, immune signaling, metabolic homeostasis, and central network activity, contributing to peripheral sensitization, impaired descending inhibitory control, and persistent changes in neural function that sustain pain in the absence of ongoing tissue injury [3,11]. Understanding these mechanisms is crucial for identifying systemic pharmacological treatments that target the underlying disease biology.

### 2.1. Central Neuroinflammation and Glial Activation

Neuroinflammation is a fundamental component of chronic pain pathophysiology [12]. Microglial cells and astrocytes within the central nervous system transition from a resting state to a reactive state following nerve damage, metabolic insult, infection, or peripheral inflammation, resulting in the secretion of proinflammatory cytokines, chemokines, and neuroactive mediators [5,13]. Activated microglial cells utilize a variety of signaling pathways, including Toll-like receptor 4, purinergic receptors P2X4 and P2X7, NF-κB, and the NLRP3 inflammasome, thereby contributing to a self-reinforcing environment of neuroinflammation that potentiates nociceptive transmission [14,15]. In contrast, astrocytes have been implicated in increased extracellular glutamate levels, changes in neurotransmitter release, and increased excitatory synaptic efficiency in spinal and supraspinal networks [16,17]. This leads to increased central sensitization even when the pathophysiological insult is cleared [3].

### 2.2. Peripheral Sensitization and Nociceptor–Immune Crosstalk

Peripheral sensitization results from complex interactions between nociceptors, immune cells, and glial components of the peripheral nervous system [18]. After nerve injury, an abundance of macrophages, mast cells, and T lymphocytes is observed around injured nerves, as well as within the dorsal root ganglion [19,20]. These cells release a variety of cytokines, growth factors, and lipids that increase the sensitization of nociceptors by enhancing the expression and activity of ion channels, such as transient receptor potential cation channel subfamily V member 1 (TRPV1) and sodium voltage-gated channel alpha subunit 9 (also known as Nav1.7), resulting in reduced thresholds for activation, increased spontaneous firing, or even ectopic firing [18,20]. Satellite glial cells within the dorsal root ganglion further enhance these inputs and communicate with sensory neurons bidirectionally, potentiating inflammation [21]. Peripheral sensitization increases nociceptive input to the spinal cord, enhances central sensitization, and contributes to the development of chronic pain [22].

### 2.3. Mitochondrial Dysfunction and Oxidative Stress

Mitochondrial dysfunction and oxidative stress are increasingly recognized as shared mechanisms across diverse chronic pain states, including neuropathic, centralized, and musculoskeletal pain conditions [23]. Overproduction of reactive oxygen species disrupts the mitochondrial membrane potential, disrupts ATP production, and activates redox-sensitive transcription factors, which drive an inflammatory cascade [23,24]. Oxidative stress enhances the excitability of neurons, contributing to the stabilization of hyperexcitable states in peripheral and central nervous system cells [24]. Oxidative stress also facilitates cellular damage, which, in turn, is hypothesized to contribute to neurodegeneration within the pain pathways [23]. Most systemic medications that are thought to be amenable to drug repositioning, such as GLP-1 receptor agonists, SGLT2 inhibitors, metformin, statins, and phosphodiesterase inhibitors, suppress oxidative stress and enhance mitochondrial function, thereby providing a potential mechanism linking metabolic modulation to analgesic responses [25,26].

### 2.4. Metabolic Dysregulation and the AMP-Activated Protein Kinase (AMPK)/Mammalian Target of Rapamycin (mTOR) (AMPK/mTOR) Axis

Metabolic dysfunction is strongly associated with chronic pain [27]. The AMPK/mTOR pathway plays an important role in regulating energy homeostasis and inflammation [27,28]. Downstream AMPK activity, which occurs in conditions such as obesity, diabetes, and low-grade chronic inflammation, increases NF-κB signaling, inflammasome activation, autophagy impairment, and neuronal hyperexcitability via mTOR activation [28]. This leads to peripheral as well as central sensitization, which may account for the close patho-epidemiological link between chronic pain conditions and metabolic dysfunction [27]. Agents that activate downstream AMPK signaling or affect mTOR activity, such as metformin and SGLT2 inhibitors, are of great interest for the treatment of chronic pain [29].

### 2.5. Integrated Opportunities for Systemic Pharmacologic Modulation

The intersection of neuroimmune activation, mitochondrial dysfunction, oxidative damage, and metabolic derangement reveals several therapeutic opportunities that are directly amenable to multiple systemic medications currently in widespread use. Drugs originally designed for endocrine, cardiovascular, and immunological purposes frequently demonstrate profound effects on glial responsiveness, inflammation, mitochondrial biology, and ion channels, which are fundamental to the pathophysiological mechanisms of chronic pain. Such non-traditional uses constitute a compelling biological rationale for evaluating pharmacological agents as potential systemic therapies for chronic pain in patients, where currently available analgesic practices are limited by low efficacy, safety issues, or other factors.

When these pathophysiologic pathways are evaluated in clinical studies, pharmacologic interventions are typically administered using standard dosing regimens approved for non-analgesic indications, with treatment duration ranging from short-term exposure to prolonged administration depending on study design. Patient populations evaluated in these studies commonly include individuals with neuropathic pain, centralized pain syndromes, migraine, fibromyalgia, inflammatory pain conditions, or pain comorbid with metabolic or neuroinflammatory disease. Accordingly, dosage, timing of administration, and patient-related factors are discussed in subsequent sections when relevant, with emphasis placed on pathophysiologic plausibility and translational context rather than prescriptive dosing recommendations. The following sections concentrate on pharmacological agents that, based on either their mechanisms of action or preliminary clinical findings, may play a role in managing chronic pain.

## 3. Repurposed Metabolic Agents with Emerging Analgesic Potential

### 3.1. GLP-1 Receptor Agonists

GLP-1 receptor agonists, commonly used to treat type 2 diabetes and obesity, exhibit potent anti-inflammatory and neuroprotective effects that extend beyond glycemic management [30]. The presence of GLP-1 receptors in neurons, microglial cells, astrocytes, and endothelial cells makes it feasible for these drugs to target multiple pathways implicated in chronic pain [31]. Experimental research has revealed that GLP-1 receptor activation can reduce microglial activation, NF-κB/JAK/STAT signaling, and decrease the production of proinflammatory cytokines, such as tissue necrosis factor (TNF)-α, interleukin (IL)-1β, and IL-6 [32]. Additionally, GLP-1 receptor agonists can alleviate oxidative stress by enhancing mitochondrial activity and reducing the production of reactive oxygen species, thereby reducing excitatory neurotransmission sensitization in the central nervous system [9]. Imaging studies on brain function have revealed a decrease in the activity of the insular cortex, which is significantly linked to chronic pain, as well as that of the anterior cingulate cortex [33]. Despite the lack of large-scale clinical research, available studies on GLP-1 receptor agonists have consistently reported improvements in neuropathic symptoms, regardless of glycemic changes [34,35]. Available human data relate primarily to diabetic peripheral neuropathy and neuropathy-associated sensory symptoms, with additional exploratory evidence in headache disorders summarized in broader reviews [34,35]. To date, clinical evidence has largely consisted of small observational cohorts demonstrating modest but consistent signals across neuropathy-related outcomes, suggesting a possible role in pain modulation, although clear analgesic benefit has not been established.

Human clinical data include a prospective observational study of 22 participants with type 2 diabetes evaluated before and after initiation of GLP-1 receptor agonist therapy (semaglutide or dulaglutide) using standard metabolic dosing [36]. Improvements were observed at 1 month in tibial nerve morphology assessed by ultrasound, with a subset demonstrating further improvement at 3 months accompanied by reduced neuropathy severity scores and improved sural sensory nerve conduction amplitude [36]. These findings indicate short-term improvement in neuropathy-related clinical measures, while definitive analgesic effects remain to be confirmed in larger controlled trials. A recent systematic review further summarizes available human and preclinical evidence across headache, neuropathic, inflammatory, and visceral pain conditions [37]. Reported clinical endpoints across available studies include neuropathy severity scores, structural nerve assessments, and neurophysiologic measures, while standardized pain intensity or functional outcomes are inconsistently reported, limiting conclusions regarding direct analgesic efficacy [36,37].

### 3.2. SGLT2 Inhibitors

SGLT2 inhibitors were initially developed to promote glycosuria in hyperglycemia; however, their metabolic and anti-inflammatory effects have prompted interest in their potential relevance to chronic pain mechanisms [38]. These agents activate AMP-activated protein kinase, suppress NF-κB signaling, and reduce mitochondrial oxidative stress, processes implicated in nociceptor hyperexcitability and neuroinflammation [39]. In preclinical models, SGLT2 inhibitors increase mitochondrial membrane potential, reduce oxidative stress in peripheral neurons, and attenuate mechanical allodynia and thermal hyperalgesia [39,40]. Improvements in endothelial function and peripheral nerve perfusion may also contribute to symptom modulation, particularly in pain states associated with microvascular dysfunction [41,42]. Available data relates predominantly to diabetic peripheral neuropathy and neuropathy-associated sensory symptoms, reflecting the populations in which these agents have been most extensively studied [40,42].

Although favorable results are available from a limited number of clinical trials, patients with diabetes who take SGLT2 inhibitors experience alleviation of neuropathic symptoms, encouraging further research on the use of these agents [40,42]. The multiple potential mechanisms of action of SGLT2 inhibitors make them ideal candidates for treating chronic pain. Clinical research is less developed, with most available data from retrospective metabolic research cohorts.

SGLT2 inhibitors have primarily been evaluated in diabetic populations using standard approved dosing regimens for glycemic control (Table 1), with treatment duration and follow-up typically spanning several weeks to a few months depending on study design [40,42]. Systematic review and meta-analysis level evidence suggests possible improvement in diabetic neuropathy-related outcomes and symptom measures in some cohorts, but the overall evidence base remains limited and is derived largely from metabolic or neuropathy-focused studies. Accordingly, reported clinical endpoints vary across studies (neuropathy symptom scores, neurophysiologic measures, and related clinical outcomes), limiting cross-study comparability. Across these studies, clinical endpoints most commonly include neuropathy symptom scores, electrophysiologic measures, and related neurologic outcomes, while standardized pain intensity, functional outcomes, and responder analyses are inconsistently reported [40,42].

### 3.3. Metformin

Metformin is a widely used medication with well-described anti-inflammatory, metabolic, and neuroprotective effects mediated primarily through activation of AMP-activated protein kinase (AMPK) [43,44]. Through AMPK signaling, metformin suppresses NF-κB-dependent inflammatory pathways, inflammasome activity, and proinflammatory cytokine production in glial and peripheral immune cells [43,44]. Metformin also modulates neuronal excitability by influencing the mTOR pathway and supporting mitochondrial function in neural tissues [45]. Preclinical studies consistently demonstrate analgesic effects of metformin in models of diabetic neuropathy, chemotherapy-induced neuropathy, and inflammation-related neuropathic pain [35,46]. Observational studies in patients with metabolic disorders treated with metformin have reported lower neuropathic symptom burden, although findings remain inconsistent [44,47]. Its favorable safety profile, low cost, and widespread availability make metformin an attractive candidate for therapeutic repurposing in chronic pain conditions.

The literature for metformin in chronic pain remains primarily observational and is derived from patients treated for metabolic indications using standard dosing regimens (Table 1) over extended treatment durations. Some observational datasets report reduced neuropathic symptom burden among metformin-exposed patients; however, confounding by indication and concomitant therapies limits causal inference, and pain-specific randomized trials are lacking. Current human data therefore support a biological basis and highlight the need for prospective studies with predefined pain-related endpoints and treatment duration [29,47].

## 4. Repurposed Anti-Inflammatory and Neuroimmune-Modulating Agents

### 4.1. Statins

Statins, widely recognized for their use in cardiovascular disease, possess anti-inflammatory and immunomodulatory properties beyond their lipid-lowering effects [48,49]. Mechanisms implicated in statin-mediated anti-inflammatory activity include suppression of key proinflammatory mediators such as TNF-α, IL-1β, and IL-6, as well as reduction in oxidative stress [48,50]. Statins may also influence ion channel activity; recent findings suggest modulation of TRPV1 channels, which play a central role in nociceptive signaling [51]. In preclinical studies of neuropathic pain, statin treatment has been associated with reduced pain-related behaviors, supporting a potential mechanistic link between statin therapy and nociceptive modulation. Existing data relate primarily to peripheral neuropathic pain, particularly diabetic peripheral neuropathy and dyslipidemia-associated neuropathic symptoms, reflecting the populations in which statins have been most extensively studied [52,53].

Observational studies have reported a decreased prevalence of neuropathic pain among patients receiving statins [52,53]. However, most available evidence derives from population-level analyses, and reported effect sizes are variable.

Statins have been evaluated in human populations primarily through observational cohorts and secondary analyses of cardiovascular trials, using standard lipid-lowering doses administered chronically over months to years [52,53]. Several population-based studies report an association between statin exposure and a lower prevalence or reduced incidence of peripheral neuropathy, particularly in patients with diabetes or dyslipidemia; however, findings are inconsistent and not derived from pain-specific randomized trials. Clinical endpoints across studies include neuropathic symptom reporting and diagnostic coding as opposed to standardized pain intensity measures, limiting conclusions regarding analgesic efficacy. Overall, human data support anti-inflammatory and neuroimmune-modulating plausibility, but definitive pain-related benefit remains unestablished [7,25,52,53].

### 4.2. Minocycline

Minocycline, a tetracycline compound with broad antimicrobial use, has been extensively investigated for its potential role in microglial modulation and neuroinflammation [8,54]. It suppresses toll-like receptor 4 signaling, inhibits proinflammatory cytokine release, reduces metalloprotease expression, and stabilizes mitochondrial function, mechanisms that are relevant to central sensitization [55,56]. In preclinical models of neuropathic pain, early treatment with minocycline has consistently reduced mechanical and thermal hypersensitivity [54]. In contrast, findings from human studies have been equivocal. Small clinical trials have reported mild or inconsistent analgesic effects, with many studies demonstrating limited benefit [8]. These discrepancies may reflect differences in treatment timing, patient populations, and underlying pain mechanisms.

Minocycline has been evaluated in small randomized or pilot clinical trials across neuropathic and centralized pain conditions, typically using antimicrobial dosing regimens administered over short treatment periods ranging from days to several weeks. Clinical outcomes have included pain intensity scores and sensory thresholds, with generally modest effect sizes. Negative or equivocal findings appear more common when treatment is initiated in established chronic pain states, underscoring the importance of timing and patient selection. Accordingly, available clinical data do not support routine use of minocycline for chronic pain but provide proof of concept for glial-targeted strategies [8,57].

### 4.3. Ibudilast

Ibudilast, a phosphodiesterase (PDE) inhibitor and glial cell suppressor, is used in Japan for asthma and post-stroke dizziness. It has been identified as a candidate for chronic pain treatment [8,58]. Its mechanism of action includes suppression of microglial and astrocytic activation, inhibition of proinflammatory cytokine production, and augmentation of cAMP signaling, which reduces excitatory neurotransmission in central pain pathways [8,58]. In preclinical models of neuropathic pain, ibudilast reduces allodynia and hyperalgesia through modulation of neuroimmune signaling [59,60]. These mechanisms provide a biological rationale for its investigation as a systemic analgesic, particularly in pain states characterized by glial activation.

Clinical evaluation of ibudilast has been limited to early-phase trials and small cohort studies, most commonly involving patients with neuropathic pain or comorbid opioid use disorder. In these studies, ibudilast was administered systemically at fixed oral doses over several weeks, with clinical endpoints including pain intensity, allodynia, and opioid-related hyperalgesia. Reported effects have been variable, with some trials demonstrating modest improvement in pain-related symptoms and others showing minimal benefit, reflecting heterogeneity in patient populations and outcome measures. While these findings support biological plausibility for glial modulation in pain, larger randomized trials with standardized pain endpoints are required [59,60].

### 4.4. Other Phosphodiesterase (PDE) Inhibitors

In addition to ibudilast, other PDE inhibitors, such as cilostazol and roflumilast, have demonstrated anti-inflammatory and neuromodulatory effects relevant to chronic pain-related conditions [61,62]. PDE inhibitors increase intracellular cAMP levels, thereby suppressing inflammatory signaling, promoting neuroprotection, and enhancing microvascular function, all of which may influence nociceptive processing [63,64]. In preclinical models, PDE inhibitors have been shown to reduce mechanical sensitization, mitigate mitochondrial dysfunction, and protect against neuronal injury associated with neuropathic and inflammatory pain states [61,62,65].

Clinical evidence for PDE inhibitors other than ibudilast in chronic pain remains sparse. Human studies have largely focused on vascular or metabolic indications using standard approved dosing, with pain-related outcomes reported as secondary or exploratory endpoints when present. Consequently, treatment duration, dosing, and outcome measures vary widely across studies, and pain-specific randomized trials are lacking. This emphasizes the need for prospective clinical trials specifically designed to evaluate pain-related endpoints [61,62,63,64,65].

## 5. Repurposed Agents with Neurologic or Autonomic Modulatory Effects

### 5.1. Low-Dose Naltrexone (LDN)

LDN has received increasing attention as a potential treatment for chronic pain due to its immunoregulatory mechanisms, which are distinct from classical opioid receptor antagonism [66,67,68]. At low doses, typically ranging from 1 to 6 mg daily, naltrexone exhibits anti-inflammatory effects through inhibition of toll-like receptor 4 (TLR4) signaling on microglial cells and macrophages [66,69]. This mechanism is associated with reduced proinflammatory cytokine release, attenuation of glial activation, and suppression of central sensitization [67,68]. Transient opioid receptor blockade may also enhance endogenous opioid activity through compensatory upregulation of opioid signaling pathways [68,69]. In fibromyalgia, complex regional pain syndrome (CRPS), and other centralized pain syndromes, small clinical studies have reported improvements in pain intensity, fatigue, and quality-of-life measures [66,70].

Clinical evaluation of LDN has primarily consisted of small randomized crossover and placebo-controlled trials, most commonly in patients with fibromyalgia. In these studies, naltrexone was administered orally at doses of approximately 3–6 mg once daily over treatment periods of 8–12 weeks. Reported outcomes included reductions in pain intensity, fatigue, and symptom severity scores, with modest but statistically significant benefits observed in some cohorts alongside substantial interindividual variability. Although these findings suggest potential benefit in selected centralized pain populations, the evidence base remains limited, and larger randomized trials with standardized pain endpoints are needed to define its clinical role [70,71,72,73,74].

### 5.2. Beta-Blockers

Beta-adrenergic antagonists, particularly propranolol and nebivolol, are systemically administered medications that may exert analgesic effects in selected pain conditions [75,76]. Chronic pain is frequently associated with increased sympathetic activity and autonomic nervous system dysregulation, which can amplify nociceptive signaling, stress responses, and maladaptive pain processing [77]. Beta-blockers reduce sympathetic outflow, modulate adrenergic receptor sensitivity, and attenuate central mechanisms involved in pain amplification [76,78,79]. In addition to their autonomic effects, certain beta-blockers demonstrate anti-inflammatory actions, reduce oxidative stress, and modulate ion channel activity in peripheral nerves [51,78]. Propranolol is well established for migraine prophylaxis and has also shown analgesic effects in pain conditions characterized by adrenergic hyperactivity, although its role in peripheral neuropathic pain remains uncertain [75,76].

Clinical studies evaluating beta-blockers for pain have primarily focused on conditions associated with autonomic dysregulation, including migraine and temporomandibular disorder. In randomized, placebo-controlled trials, propranolol administered at standard antihypertensive or migraine-prophylactic doses over several weeks has been associated with reductions in pain intensity and symptom frequency in select populations. In contrast, evidence supporting analgesic efficacy in peripheral neuropathic pain is limited, and reported outcomes vary according to pain phenotype and degree of autonomic involvement. Therefore, it is reasonable to assume a benefit in sympathetically mediated or centrally modulated pain states, instead of across neuropathic pain conditions more broadly [75,76,77,78,79,80].

### 5.3. Cannabinoid-Modulating Pharmaceuticals

Pharmaceuticals that interact with the endocannabinoid system, particularly those targeting the cannabinoid receptor type 2 (CB2), have potential relevance for chronic pain management [81,82]. In contrast to CB1 receptors, which are primarily associated with psychotropic effects, CB2 receptors are predominantly expressed on immune cells, microglia, and peripheral nociceptors, where they regulate cytokine release, neuroimmune signaling, and neuronal sensitization during inflammation [83,84]. Preclinical studies demonstrate that selective CB2 agonists and endocannabinoid modulation reduce microgliosis, proinflammatory activity, and mechanical and thermal hyperalgesia in models of neuropathic pain [82,85]. Early human studies and observational data suggest acceptable safety and tolerability profiles; however, analgesic efficacy has not been systematically evaluated in adequately powered trials [81,83,84]. The relative absence of psychotropic effects makes CB2-focused agents attractive candidates for further investigation in chronic pain.

Available evidence for CB2-modulating agents in chronic pain remain limited and are largely derived from early-phase or exploratory studies designed to assess safety, tolerability, and pharmacodynamic effects. In these studies, systemically administered CB2-selective or peripherally acting cannabinoid agents were evaluated over short treatment durations, with pain-related outcomes included as secondary or exploratory endpoints when reported. While these data support biological plausibility and a favorable safety profile, definitive conclusions regarding analgesic benefit cannot yet be drawn due to small sample sizes, short follow-up periods, and heterogeneity in outcome measures [81,82,83,84,85,86,87,88,89].

## 6. Integrative Mechanistic Framework Across Repurposed Drug Classes

### 6.1. Convergent Mechanisms Linking Repurposed Drugs to Chronic Pain Modulation

Across pharmacologic classes, multiple systemic medications originally developed for different indications converge on shared biologic pathways implicated in chronic pain. Agents, including GLP-1 receptor agonists, SGLT2 inhibitors, metformin, statins, minocycline, ibudilast, low-dose naltrexone, beta-blockers, and cannabinoid-modulating medications, address fundamental components of pain chronification through distinct therapeutic pathways. In aggregate, these medications target shared neuroimmune mechanisms, including reductions in microglial and astrocytic activation and downstream inflammatory signaling that sustains central sensitization [8,90,91]. Several agents target the mechanisms of oxidative damage, thereby restoring cellular metabolic balance within neurons, astrocytes, and microglial cells [39,43,50,92]. Additional mechanisms, including ion channel modulation, improvements in endothelial or microvascular function, and modulation of autonomic tone, may further attenuate nociceptive amplification [41,51]. Importantly, these convergent mechanisms are supported by human clinical observations across multiple repurposed drug classes, although the strength of evidence varies. In clinical studies, modulation of neuroimmune or metabolic pathways has been associated with changes in symptom burden and pain-related quality-of-life measures over treatment periods of weeks to months.

### 6.2. Mechanism-Based Matching to Pain Phenotypes

**Figure 2:**
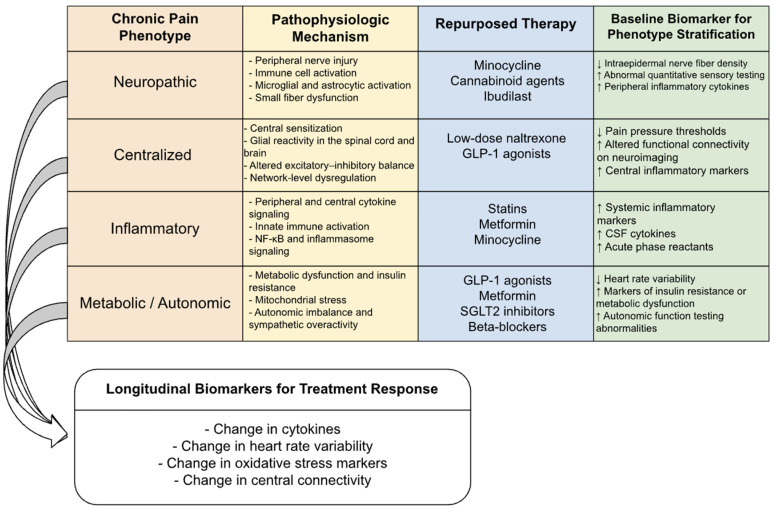
Flowchart illustrating phenotype-based stratification of chronic pain, corresponding pathophysiologic mechanisms, candidate repurposed systemic therapies, baseline biomarkers for patient selection, and longitudinal biomarkers for monitoring treatment response.

The heterogeneity of chronic pain suggests that there is no single, universally effective treatment; therefore, existing repurposed pharmacological classes may facilitate the development of phenotype-specific treatment modalities. Patients presenting with neuroimmune-mediated conditions such as exaggerated glial responses, fibromyalgia, CRPS, or neuropathic pain following nerve injury most likely benefit from medications such as minocycline, ibudilast, LDN, and statins with known anti-inflammatory effects [8,10,93]. Those with exaggerated metabolic dysfunction, mitochondrial dysfunction, and comorbidities of type 2 diabetes mellitus may find existing GLP-1 receptor agonists, SGLT2 inhibitors, or metformin, which aim to rebalance cellular energy status, thereby suppressing oxidative stress [7,25,43]. In contrast, patients with sympathetically modulated, anxiety-mediated, or other types of sympathetically conditioned profiles would most likely benefit from beta blockers and autonomic-modulatory medications [10,57], and so on. Cannabinoid modulatory agents would theoretically work best in patients with exaggerated immune responses, peripheral sensitization, and/or overall generalized inflammation [71,81]. Data supporting mechanism-based matching remains preliminary and is largely derived from condition-specific studies and subgroup analyses. Reported outcomes suggest that agents targeting neuroimmune, metabolic, or autonomic pathways may preferentially benefit select pain phenotypes over treatment periods of weeks to months; however, formal stratification by mechanistic profile is rarely performed, limiting generalizability. This phenotype-based stratification approach and its associated candidate therapies and biomarkers are summarized in Figure 2.

### 6.3. Integration with Existing Multimodal and Interventional Strategies

Repurposed systemic drugs may also support or potentiate existing multimodal chronic pain therapies. Their anti-inflammatory and neuro-immunomodulatory properties may contribute additively to the restoration of physical, psychological, and biological function [72,73]. In perioperative and interventional settings, medications that attenuate neuroinflammation, thereby potentially lowering the likelihood of transition from acute to chronic pain, are of significant clinical interest [74,80]. Preliminary evidence further suggests potential synergistic effects between interventional medications that mitigate neuroinflammatory and oxidative stress, together with non-invasive brain stimulation, spinal cord stimulation, and peripheral nerve stimulation [86,87]. When designed to focus on neuroimmune mechanisms implicated in device failure, these medications may enhance the long-term efficacy of interventional modalities [73,88]. This convergence of mechanism-focused pathways signifies the broad potential utility of repurposed medications within a tailored, mechanism-based, treat-to-target paradigm for chronic pain management. It is important to emphasize that the current understanding of these mechanisms remains incomplete. Consequently, repurposed agents should be considered complementary and synergistic to established modalities, ensuring they are integrated without implying replacement of existing therapies. In clinical contexts, repurposed systemic agents have most often been evaluated as adjuncts to multimodal or interventional therapies, with outcomes including symptom burden, functional measures, or treatment durability. Available human data are heterogeneous and typically short-term, underscoring the need for standardized endpoints when assessing synergy within integrated treatment strategies.

## 7. Current Clinical Evidence and Translational Considerations

### 7.1. Overview of Emerging Human Evidence

Although these repurposed systemic medications were not developed initially as analgesics, there is a growing body of clinical evidence that indicates that many of these medications may meaningfully interact with pain pathways [89,94]. Observational, retrospective, case series, and small pilot studies have provided promising evidence of the analgesic properties of several repurposed systemic agents [8]. For instance, patients treated with repurposed systemic agents for various metabolic conditions have demonstrated improvements in neuropathic symptoms, regardless of glycemic control [34,36,40]. Additionally, patients taking statins have shown a reduced incidence of certain neuropathic illnesses [95,96]. Recent studies have demonstrated various medications offering significant symptomatic relief in patients with fibromyalgia, complex regional pain syndrome, and a host of central illnesses [66,67,70]. Although these findings are encouraging, the full scope of available evidence is not comprehensively supported, with results varying from trial to trial. Several agents, including minocycline, statins, and beta-blockers, demonstrate inconclusive or null findings in controlled trials. Across reported human studies, repurposed agents have generally been administered at standard approved doses or established low-dose regimens over treatment periods ranging from several weeks to a few months. Reported clinical outcomes most commonly include changes in neuropathic symptom burden, fatigue, and pain-related quality-of-life measures, as opposed to consistent reductions in pain intensity across conditions.

### 7.2. Safety, Tolerability, and Comparative Advantages

The most compelling aspect of exploring repurposed systemic medications for chronic pain is their proven safety profile [6,97]. Medications, such as metformin, GLP-1 receptor agonists, SGLT2 inhibitors, statins, and beta-blockers, have been administered to large populations for many years and even decades, with robust safety profiles [98,99,100]. The predictability of safety, lack of abuse potential, or physiological dependence potential makes such medications attractive alternatives, even add-on medications, to current analgesics, especially with increased concerns about prolonged opioid use and unresolved concerns regarding the efficacy of gabapentinoids and tricyclic antidepressants in patients with chronic pain-related conditions [101,102]. Furthermore, the use of such medications provides patients with multiple conditions, including chronic conditions, such as diabetes, coronary heart disease, obesity, and hyperlipidemia, with a chance to gain various potential benefits from a single medication [101,102]. Anti-inflammatory and neuroimmune-modulatory agents such as minocycline, ibudilast, and LDN are also relatively safe at the doses used [8,67]. The multiple factors that make these agents amenable to further evaluation in patients with chronic pain were significantly reinforced. The dosing strategies employed in pain-related clinical studies have largely mirrored approved or well-established regimens, contributing to favorable tolerability profiles and low rates of treatment discontinuation. However, because these agents are being considered outside their approved indications, safety and tolerability profiles may differ in chronic pain populations, underscoring the need for cautious patient selection and pain-specific clinical evaluation.

### 7.3. Implementation Challenges and Opportunities for Precision Medicine

Despite the encouraging mechanism-based and very early clinical findings, specific implementation issues need to be clarified before repurposed systemic medications can be generally introduced within treatment models for chronic pain. The lack of large, high-quality randomized clinical trials means that no definitive conclusions can currently be drawn regarding the efficacy, dosing, and patient population [6,8,37]. The phenotypes of chronic pain are diverse, with varying degrees of neuroimmune activation, metabolic dysfunction, psychosocial profiles, and/or coexisting diseases [1,10,103]. This is a reminder that precision medicine, which connects specific, distinct mechanisms with particular biological or clinical archetypes, is a priority [37,103]. Biomarker-driven stratification, focusing on inflammatory, metabolic, neuroimaging, and quantitative sensory testing profiles, may assist in this regard [104,105]. In addition, when combining these medications with existing multimodal treatment approaches, particular attention is required to address the issues of polypharmacy, pharmacokinetic interactions, and shared metabolic routes [106,107]. Despite these difficulties, the increased mechanistic insights into chronic pain mechanisms, combined with the expansion of patient-level databases, provide an opportunity to design personalized, tailored treatment plans with a focus on repurposing systemic medications. Existing clinical studies are typically short in duration and heterogeneous in outcome selection, limiting conclusions regarding long-term efficacy or optimal treatment duration. As a result, current evidence primarily informs feasibility and hypothesis generation.

## 8. Future Directions and Research Gaps

### 8.1. Advancing Mechanistically Informed Clinical Trials

Although there is a sound rationale from a mechanistic perspective as well as promising preliminary data, the translation of these agents into clinical settings for the treatment of chronic pain is still in its infancy. The majority of the current literature has been generated from observational research, pilot studies, or findings from patients with non-pain-related disorders treated in a similar population [6,97]. The lack of a solid, randomized controlled study focusing on the use of phenotypic assessment to describe analgesic responses is a significant barrier to the translation of existing agents for chronic pain treatment. Not only should randomized research concentrate on a phenotypic description with predefined metrics, but it should also consider a mechanistically grounded endpoint, such as a description of inflammatory indices, imaging, and even quantitative descriptions with phenotypic indications for sensory perceptions [108,109]. In addition, these studies should examine the pharmacodynamic properties of the treatment effect, which may be significantly different from what has been observed in coronary diseases [109,110].

### 8.2. Integrating Repurposed Medications into Precision Pain Medicine

The heterogeneity of chronic pain is a significant barrier to the development of therapeutic agents; however, targeted mechanism-based approaches can be applied. Off-label agents are characterized by distinct mechanisms that may be particularly advantageous for certain types of pain. The goal of future research should be to develop models that combine molecular, metabolic, neuroimmune, and psychosocial information to identify patients who would optimally respond to a particular treatment [111,112,113]. Composite analyses that consider clinical factors together with biomarkers, such as cytokine profiles, mitochondrial biomarkers, and/or metabolic factors, may facilitate the development of stratified treatment approaches [108,111,113,114]. Adaptive trial designs that enable real-time adjustments based on biomarkers and/or disease activity may also facilitate the identification of agents with the most efficacious mechanism for a specific subtype [115,116]. The use of repurposed systemic agents is becoming increasingly integral to personalized models of pain management as precision pain medicine rapidly develops.

### 8.3. Exploring Combination Strategies with Neuromodulation and Lifestyle Interventions

Potential research directions include exploring a combination therapy approach that involves the use of repurposed systemic medications in conjunction with nonpharmacological treatments. Neuromodulatory modalities, such as peripheral nerve stimulation, spinal cord stimulation, transcranial magnetic stimulation, and transcutaneous electric nerve stimulation, focus on neural network activity and descending modulation pathways [117,118,119]. The use of repurposed medications that are thought to reduce neuroinflammation and/or address mitochondrial dysfunction might make such modalities more responsive to treatment, thereby providing a more favorable neurobiological environment for modulation [120,121]. Similarly, nonpharmacological interventions, such as structured exercise, weight reduction, sleep modulation, and dietary changes, may synergistically interact with the metabolic and anti-inflammatory properties of medications like metformin, GLP-1 receptor agonists, and/or SGLT2 inhibitors.

## 9. Conclusions

Despite available treatments, chronic pain remains difficult to manage because of biological complexity. Advances in understanding neuroimmune activation, metabolic dysfunction, mitochondrial impairment, and autonomic dysregulation have identified mechanistic targets relevant to chronic pain. These insights support re-evaluation of systemic medications developed for cardiometabolic, immunologic, or neurologic indications for potential analgesic use. Repurposed agents, including GLP-1 receptor agonists, SGLT2 inhibitors, metformin, statins, minocycline, ibudilast, low-dose naltrexone, beta-blockers, and cannabinoid-modulating therapies, intersect with pathways involved in chronic pain pathophysiology. Their safety records and clinical use provide a basis for investigation, although clinical evidence remains limited. At present, these agents are best considered adjuncts under study rather than as established treatments. Future studies should focus on mechanism-based trial design, biomarker-guided patient selection, and assessment of combination approaches with neuromodulation and lifestyle interventions to define the role of repurposed systemic agents in chronic pain research.

## Figures and Tables

**Table 1:** Summary of repurposed systemic pharmacologic agents, their pain-relevant mechanisms, and emerging clinical evidence across chronic pain phenotypes. Abbreviations: ↑ indicates upregulation, activation, or increase in the listed pathway or process; ↓ indicates downregulation, inhibition, or reduction.

Drug Class (Examples)	Primary Approved Indications	Pain-Relevant Mechanisms	Best-Supported Pain Phenotypes/Conditions	Human Evidence Level	Key Safety/Contraindications	Practical Notes	Representative Clinical Dosing/Administration (Examples)
GLP-1 Receptor Agonists (semaglutide, liraglutide)	Type 2 diabetes; obesity	↓ Microglial activation ↓ Pro-inflammatory cytokines (TNF-α, IL-1β, IL-6) ↓ Oxidative stress ↑ Mitochondrial efficiency	Diabetic neuropathy Neuropathic pain Centralized pain states	Prospective observational cohorts; small non-randomized studies	GI intolerance (nausea, vomiting) Delayed gastric emptying	Typical metabolic dosing; analgesic effects appear independent of glycemic control	Semaglutide 0.25–1.0 mg weekly; liraglutide 0.6–1.8 mg daily
SGLT2 Inhibitors (empagliflozin, dapagliflozin)	Type 2 diabetes; heart failure; CKD	↓ Oxidative stress ↑ Mitochondrial membrane potential ↓ Neuroinflammation ↑ Microvascular perfusion	Diabetic neuropathy Metabolic-associated pain	Retrospective observational cohorts; small pilot studies (neuropathy outcomes)	Euglycemic ketoacidosis risk Volume depletion	No established analgesic dosing; data largely retrospective	Empagliflozin 10–25 mg daily; dapagliflozin 5–10 mg daily
Metformin	Type 2 diabetes; insulin resistance	AMPK activation mTOR inhibition ↓ NF-κB signaling ↑ Mitochondrial integrity	Diabetic neuropathy Chemotherapy-induced neuropathy Metabolic pain phenotypes	Observational cohort studies; extensive preclinical data; no pain-specific RCTs	GI intolerance Contraindicated in advanced renal failure	Widely available; low cost; favorable safety profile	500–2000 mg/day orally in divided doses
Statins (atorvastatin, simvastatin)	Hyperlipidemia; cardiovascular disease	↓ Cytokine production ↓ Oxidative stress Modulation of TRP channels	Neuropathic pain Metabolic-associated pain	Observational population-based studies; mixed findings	Myopathy (rare) Liver enzyme elevation	Analgesic effects likely pleiotropic, not lipid-dependent	Atorvastatin 10–40 mg/day; simvastatin 10–40 mg/day
Minocycline	Antimicrobial therapy	Inhibition of microglial activation ↓ Pro-inflammatory mediators Mitochondrial stabilization	Early neuropathic pain Neuroimmune-mediated pain	Small RCTs; largely negative or mixed	Dizziness, pigmentation Long-term use limitations	Timing critical; limited benefit in established chronic pain	100–200 mg/day orally
Ibudilast/PDE Inhibitors	Asthma; post-stroke dizziness (Japan)	↑ cAMP signaling ↓ Microglial and astrocyte activation ↓ Neuroinflammation	Neuropathic pain Glial-mediated pain states	Early-phase trials; small cohorts	GI symptoms Limited global availability	Experimental outside select regions	40–60 mg/day in early-phase clinical trials
Low-Dose Naltrexone (LDN)	Off-label	TLR4 inhibition ↓ Microglial reactivity Modulation of endogenous opioid tone	Fibromyalgia CRPS Centralized pain syndromes	Small crossover RCTs	Sleep disturbance (early) Minimal systemic toxicity	Typical dose 1–5 mg nightly; well tolerated	1–5 mg nightly
β-Blockers (propranolol, nebivolol)	Hypertension; arrhythmias; migraine prophylaxis	↓ Sympathetic overactivity ↓ Stress-mediated pain amplification Anti-inflammatory effects	Migraine Sympathetically maintained pain	Small trials; observational	Bradycardia Asthma (non-selective agents)	Best suited for patients with autonomic dysregulation	Agent- and indication-dependent; propranolol commonly 40–160 mg/day
Cannabinoid (CB2-Modulating) Agents	Various (region-dependent)	↓ Immune cell activation ↓ Peripheral sensitization Minimal psychotropic effects (CB2)	Neuropathic pain Inflammatory pain	Preclinical; early human studies	Regulatory limitations Limited long-term data	CB2-selective agents preferred over CB1	No standardized clinical dosing; investigational

## Data Availability

No new data were created or analyzed in this study.
